# The Difference between the Two Representative Kampo Formulas for Treating Dysmenorrhea: An Observational Study

**DOI:** 10.1155/2016/3159617

**Published:** 2016-02-24

**Authors:** Tetsuhiro Yoshino, Kotoe Katayama, Yuko Horiba, Kaori Munakata, Rui Yamaguchi, Seiya Imoto, Satoru Miyano, Hideki Mima, Kenji Watanabe, Masaru Mimura

**Affiliations:** ^1^Center for Kampo Medicine, Keio University School of Medicine, 35 Shinanomachi, Shinjuku-ku, Tokyo 160-8582, Japan; ^2^Human Genome Center, The Institute of Medical Science, The University of Tokyo, 4-6-1 Shirokanedai, Minato-ku, Tokyo 108-8639, Japan; ^3^SFC Laboratory, Keio University, 5322 Endo, Fujisawa, Kanagawa 252-0882, Japan; ^4^Division of Health Medical Data Science, Health Intelligence Center, The Institute of Medical Science, The University of Tokyo, 4-6-1 Shirokanedai, Minato-ku, Tokyo 108-8639, Japan; ^5^School of Engineering, The University of Tokyo, 7-3-1 Hongo, Bunkyo-ku, Tokyo 113-8656, Japan; ^6^Faculty of Environment and Information Studies, Keio University, 5322 Endo, Fujisawa, Kanagawa 252-0882, Japan; ^7^Department of Neuropsychiatry, Keio University School of Medicine, 35 Shinanomachi, Shinjuku-ku, Tokyo 160-8582, Japan

## Abstract

In Kampo medicine, two different formulas are effective for treating dysmenorrhea—tokishakuyakusan and keishibukuryogan; however, the criteria by which specialists select the appropriate formula for each patient are not clear. We compared patients treated with tokishakuyakusan and those with keishibukuryogan and proposed a predictive model. The study included 168 primary and secondary dysmenorrhea patients who visited the Kampo Clinic at Keio University Hospital. We collected clinical data from 128 dysmenorrhea patients, compared the two patient groups and selected significantly different factors as potential predictors, and used logistic regression to establish a model. An external validation was performed using 40 dysmenorrhea patients. Lightheadedness, BMI < 18.5, and a weak abdomen were significantly more frequent in the tokishakuyakusan group; tendency to sweat, heat intolerance, leg numbness, a cold sensation in the lower back, a strong abdomen, and paraumbilical tenderness and resistance were more frequent in the keishibukuryogan group. The final model fitted the data well. Internally estimated accuracy was 81.2%, and a leave-one-out cross-validation estimate of accuracy was 80.5%. External validation accuracy was 85.0%. We proposed a model for predicting the use of two Kampo formulas for dysmenorrhea, which should be validated in prospective trials.

## 1. Introduction

Dysmenorrhea is the most common gynecological disorder in women, regardless of age and nationality [1]. Patients with dysmenorrhea have strong lower abdominal or lower back pain that begins during or just before the menstrual period. Dysmenorrhea is thought to be caused by an excess or imbalance of prostanoids, and possibly other eicosanoids, released from the endometrium during menstruation. As a result, the uterine basal tone increases, with frequent and dysrhythmic contraction. Pain is induced by uterine hypercontractility, reduced uterine blood flow, and increased peripheral nerve hypersensitivity [2].

The standard treatment for dysmenorrhea is nonsteroidal anti-inflammatory drugs (NSAIDs) or oral contraceptives (OCs) [3, 4]. Up to 30% of patients, however, do not respond sufficiently to NSAIDs, and 10% to 20% respond to neither NSAIDs nor OCs [1]. Furthermore, NSAIDs are contraindicated in patients with a peptic ulcer or gastritis. OCs are contraindicated in those with any thrombotic predisposing factor, breast cancer, migraine with aura, or pregnancy. For these reasons, various alternative treatments have been examined, such as acupressure, vitamin B1, vitamin E, use of a hot pack, transcutaneous electrical nerve stimulation, and behavioral interventions [5].

Kampo, Japanese traditional medicine, is a leading alternative medicine [5, 6] and is popular in Japan, particularly for treating women's health issues. Two Kampo formulas are commonly used for treating dysmenorrhea [7, 8]—tokishakuyakusan and keishibukuryogan—and both have been shown to be effective in randomized placebo-controlled trials [9, 10]. In the Japanese national health insurance system, both formulas are indicated for dysmenorrhea and other gynecological conditions, including irregular menstruation, menopause, and infertility.

Kampo formulas are prescribed according to traditional pattern-based diagnosis [11], which is used in addition to Western diagnosis [12]. In Kampo medicine, pattern diagnosis refers to the unique clinical classification of the patient, which takes into account symptoms, general constitution, and other factors. The patient is differentially diagnosed with chronic health conditions, including dysmenorrhea, on the basis of disharmony in any of the following areas: the eight categories (excess-deficiency, heat-cold, interior-exterior, and yin-yang) and body constituents (qi, blood, and fluid) [13]. Tokishakuyakusan is traditionally prescribed for patients diagnosed with “deficiency,” “cold,” “interior,” “yin,” “blood deficiency,” and “fluid disturbance” [10], while keishibukuryogan is used for patients diagnosed with “excess,” “tangled heat and cold,” “interior,” “yang,” and “blood stasis.”

However, pattern diagnosis in traditional medicine is a subtle art; it takes years to master the skills required to choose the appropriate formulas, and to our knowledge, it has not yet been reported whether the prescription of Kampo formulas by specialists can be predicted without knowledge of traditional pattern diagnosis. Moreover, it is not known how subjective symptoms and objective findings differ between patients who are prescribed the different Kampo formulas.

In this study, we compared the subjective symptoms and objective findings in patients prescribed tokishakuyakusan with those in patients prescribed keishibukuryogan and used this information to derive a model that can predict the selection of either of the two formulas by specialists in Kampo medicine.

## 2. Methods

### 2.1. Patient Enrollment

This observational study included primary and secondary dysmenorrhea patients who were first-time visitors to the Kampo Clinic at Keio University Hospital, between May 2008 and December 2015. All patients were treated with either of the two formulas—tokishakuyakusan or keishibukuryogan. Patients who were treated with both formulas were excluded. Patients over 50 years of age were also excluded. The Institutional Review Board at Keio University School of Medicine approved this study.

#### 2.1.1. Comparison and Model-Development Analysis

In this analysis, we included patients who made their first visit between May 2008 and March 2013. Patients who were prescribed tokishakuyakusan were included in the “TSS” group, and those who were prescribed keishibukuryogan were in the “KBG” group. We used a browser-based questionnaire during this part of the study; the questionnaire is explained in detail in Section 2.2.

#### 2.1.2. External Validation Analysis

The predictive model was validated using a different data set (the external validation group), obtained from patients who made their first visit to Kampo Clinic at Keio University Hospital between April 2013 and December 2015. We did not use the browser-based questionnaire system during this part of the study. The systems used in the medical interview were reviewed using a paper-based questionnaire, and this database was entirely separate from that used in the comparison and model-development analysis; however, the items in the questionnaire were identical.

### 2.2. Data Collection

In 2008, Keio University first introduced a browser-based questionnaire to collect information about patients' subjective symptoms, as well as their age, sex, body mass index (BMI), lifestyle, Western diagnosis (based on the international classification of diseases (ICD-10)), traditional medicine pattern-based diagnosis (based on ICD-11 beta version) [11], and Kampo formulas prescribed by Kampo specialists. Kampo specialists from representative Universities and Kampo institutions in Japan (Keio University, Chiba University, Toyama University, Jichi Medical University, Tokyo Women's Medical University, Tohoku University, Kameda Medical Center, and Aso Iizuka Hospital) prepared the questionnaire after repeated discussions. Using this questionnaire, which comprises 128 binary questions, we collected information about our patients' subjective symptoms, as described in our previous report [14].

BMI was assessed in 2 ways: as a sequential variable (crude BMI) and as binary variables: “slim” (yes/no) and “obese” (yes/no). Patients with a BMI < 18.5 were considered slim, and those with a BMI ≥ 25 were considered obese, as defined by the Japan Society for the Study of Obesity.

Data regarding each objective factor, including abdominal and tongue findings, were also collected as binary variables. Specifically, abdominal findings included nine items; one of these—abdominal strength—contained three mutually exclusive categories: weak, intermediate, and strong. Here, however, we used binary variables to code the abdominal strength: “weak abdomen” (yes/no) and “strong abdomen” (yes/no). Abdominal strength is determined by abdominal examination, whereby the doctor presses the palm of his/her hand onto the patients' abdomen to assess both the degree of resistance offered by the muscles and the thickness of the abdominal muscle wall and fat [15]. Other abdominal findings were also expressed in binary form, namely, epigastric discomfort, palpable abdominal aortic pulsation, hypochondrial resistance and discomfort, splashing sound in the epigastric region, paraumbilical tenderness and resistance, rectus muscle tension, weakness of the lower abdomen, and abdominal distension. Tongue findings included teeth marks on the edges of the patient's tongue and dilatation of the sublingual veins.

### 2.3. Comparison of Tokishakuyakusan with Keishibukuryogan

We compared each subjective and objective item between the TSS and KBG groups. We used Fisher's exact test for comparison of binary variables and Wilcoxon's rank sum test and two-sample *t*-tests for continuous variables items, such as age and crude BMI. Missing data were ignored in the tests.

### 2.4. A Predictive Model for Prescription of the Two Kampo Formulas by Specialists

#### 2.4.1. Selection of Potential Predictor Variables

We used variables with a *p* value < 0.05 in the analyses detailed in Section 2.3 as potential variables that could be used to predict which Kampo medicine would be prescribed. BMI had a *p* value < 0.05, but this information was missing for several patients; we therefore replaced the missing BMI data with the overall mean BMI during the model-development analysis.

#### 2.4.2. Model-Fitting Procedure

We applied logistic regression to the 128 data points from the TSS and KBG groups [16]; the KBG group was designated as 1, and the TSS group as 0. Using logistic regression analysis, we calculated the probability of the patient belonging to the KBG group; *p* > 0.5 indicated that the patient was predicted to belong to the KBG group, and *p* < 0.5 that the patient was predicted to belong to the TSS group. We then performed a univariate analysis on the potential predictive variables, followed by a multivariate analysis. The model that contained all the potential predictive variables was considered the full model. To measure the effect size of each predictive variable, we computed the odds ratio (OR).

However, to avoid overfitting the predictive model, the predictive variables needed to be selected more strictly, which we achieved using the Akaike information criterion (AIC) [17]. We started with the full model and challenged all possible models; the model with the lowest AIC was considered the final model.

The variance inflation factor (VIF) was used to monitor multicollinearity. We also evaluated interactions between predictor variables in the final model by including interaction terms along with main-effect terms. None of the interactions were found to be significant, and they are not discussed further in this paper.

### 2.5. Internal and External Validations of the Final Model

Calibration of the model was assessed using the area under the receiver operating characteristic curve (AUC) and the Hosmer-Lemeshow test [18]. An AUC > 0.80 and a *p* value > 0.05 in the Hosmer-Lemeshow test were considered acceptable values. The final model was internally validated by leave-one-out cross-validation. We also externally validated the final model by applying it to the external validation group's data set.

### 2.6. Statistical Analyses

All statistical analyses were conducted using R software version 3.1.1 (The R Foundation for Statistical Computing; July 10, 2014; see also: http://www.r-project.org/). We used “glm” [19] from the package “stats,” as well as the packages “DAAG” [20], “pROC” [21], and “ResourceSelection” [22]. Data are shown as mean ± standard deviation. We used a significance level of 5% for all tests but made no adjustment for multiple testing.

## 3. Results

### 3.1. Participant Information

We assessed the eligibility of 290 dysmenorrhea patients—222 patients for the comparison and model-development analysis and 68 patients for the external validation analysis.

**Figure 1:**
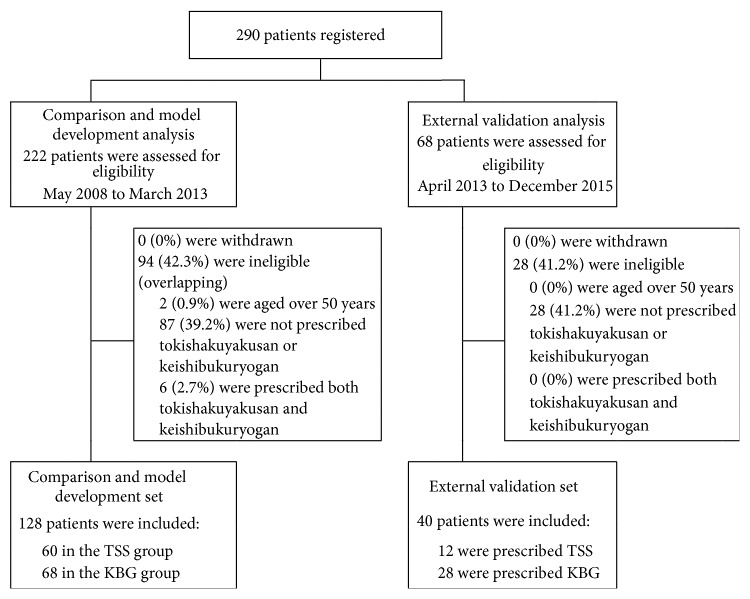
Patients' flow chart. Patients who were prescribed tokishakuyakusan only were included in the “TSS” group, and those who were prescribed keishibukuryogan only were included in the “KBG” group.

Among the 222 candidate patients for the comparison and model-development analysis, 127 had been prescribed two or more formulas (a total of 356 formulas were prescribed; Table 1). Tokishakuyakusan and keishibukuryogan were the most frequently used formulas, and 135 patients (61%) were prescribed either or both of these. None of the patients withdrew from the study. We excluded two patients who were aged over 50 years and six patients who were prescribed both tokishakuyakusan and keishibukuryogan or related formulas. Finally, we used data from 128 patients in the comparison and model-development analysis, comprising 60 who were prescribed only tokishakuyakusan (TSS group) and 68 who were prescribed only keishibukuryogan (KBG group; Figure 1: the comparison and model-development set).

Of the 68 candidate patients for the external validation analysis, 28 were excluded because they were not prescribed either tokishakuyakusan or keishibukuryogan. The data from the remaining 40 patients were used for external validation of the final model (Figure 1: the external validation set).

Table 2 summarizes the characteristics of the patients included in the two analyses. The frequency of OC use and diagnosed diseases were significantly higher in the external validation set than in the comparison and model-development set.

### 3.2. Comparison between the Characteristics of the TSS and KBG Groups

We compared the characteristics of patients in the TSS group with those of patients in the KBG group (Table 3). The BMI was significantly lower in the TSS group; correspondingly, the binary variable “slim” was significantly more frequently present. Endometriosis or adenomyosis, which leads to secondary dysmenorrhea, was found in 13.3% of TSS patients and in 22.1% of KBG patients; however, this was not significantly different. The remainder of the patients in each group was diagnosed with primary dysmenorrhea.

Five subjective symptoms and three objective findings significantly differed between the TSS and KBG groups (Table 4; Appendix Table, at Supplementary Material available online at http://dx.doi.org/10.1155/2016/3159617). Lightheadedness was more frequent in patients in the TSS group; tendency to sweat, heat intolerance, leg numbness, and a cold sensation in the lower back were more frequent in patients in the KBG group. A weak abdomen was more frequent in the TSS group, whereas a strong abdomen, as well as paraumbilical tenderness and resistance, was more frequent in the KBG group. There was no significant difference between the two groups in terms of the other 123 subjective symptoms, seven abdominal findings, or two tongue findings.

### 3.3. A Predictive Model for Prescription of the 2 Kampo Formulas by Specialists

**Figure 2:**
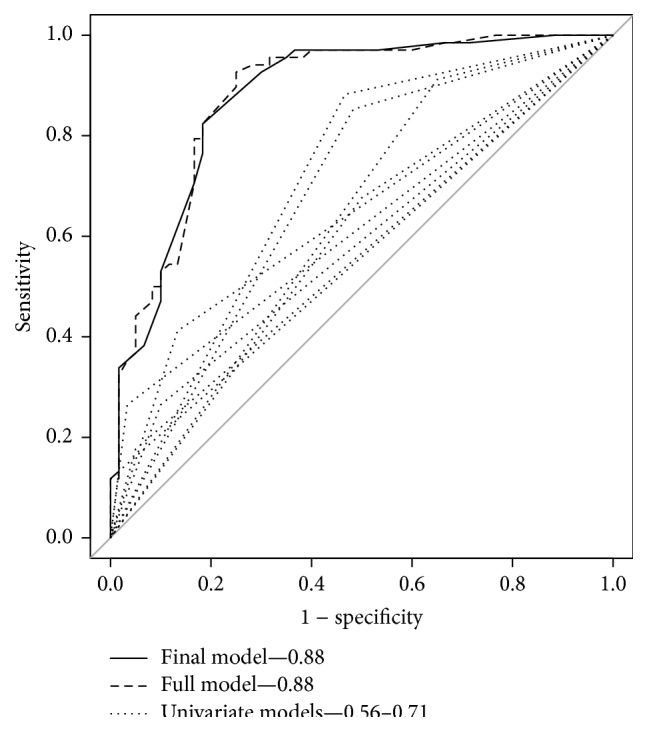
Model calibration using a receiver operating characteristic curve. The full model (broken line) included 9 predictive variables, and the final model (black line) included 7 predictive variables. Each univariate model was drawn using dotted lines. The final model had an area under the curve of 0.88 (95% CI: 0.81–0.93), and the full model had an area under the curve of 0.88 (95% CI: 0.82–0.94). The univariate models had areas under the curve of 0.56–0.71 (95% CI: not shown).

We performed univariate analyses of the five subjective symptoms and three abdominal findings that had shown a significant difference between the two groups, as well as of the variable “slim” (Table 5: univariate). We included a categorical variable “slim,” rather than the continuous variable crude BMI, as linearity cannot be achieved on the logit scale when using crude BMI. We calculated AIC and AUC for each univariate model; all models had AIC > 150 and AUC < 0.8 (Figure 2: univariate models).

We developed the full model using these nine potential predictive variables. The AIC for the full model was 127.9, and the AUC was 0.88 (95% CI: 0.82–0.94, Figure 2: full model). The Hosmer-Lemeshow test indicated that the full model fitted the comparison and model-development set (*p* = 0.1982).

After challenging all possible models, four subjective symptoms and three abdominal findings were included in the final model. The AIC for this model was 125.1, which was lower than that of the full model (Table 5: multivariate). None of the VIF values exceeded 2.0; thus, there was no collinearity in the model.

### 3.4. Internal and External Validations of the Final Model

The AUC was computed as 0.88 (95% CI: 0.81–0.93, Figure 2: final model). The Hosmer-Lemeshow test indicated that the final model fit the comparison and model-development set (*p* = 0.2519) better than did the full model. The internal estimate of accuracy of the final model was 81.2%, and the leave-one-out cross-validation estimate of accuracy was 80.5% (Table 6: internal validation). When we applied this final model to the set of 40 external validation analysis patients, we found a proper prediction rate of 85.0% (Table 6: external validation).

## 4. Discussion

Here, we have reported the differences in both subjective symptoms and objective findings between patients who had been prescribed tokishakuyakusan and those who had been prescribed keishibukuryogan. We extracted five subjective symptoms and four objective findings that were significantly different between these two groups. These items are compatible with the traditional medicine pattern diagnosis for each Kampo formula. Tokishakuyakusan is used for patients diagnosed with a “deficiency,” “cold,” “interior,” “yin,” “blood deficiency,” and “fluid disturbance” pattern. From among these selected factors, a lower BMI and weak abdomen indicate a “deficiency” and a “yin” pattern. Lightheadedness indicates a “blood deficiency” or a “fluid disturbance” pattern. Conversely, keishibukuryogan is used for patients diagnosed with an “excess,” “tangled heat and cold,” “interior,” “yang,” and “blood stasis” pattern. Higher BMI and a strong abdomen indicate an “excess” and a “yang” pattern. A tendency to sweat, heat intolerance, and a cold sensation in the lower back indicate a “tangled heat and cold” pattern. Leg numbness, as well as paraumbilical tenderness and resistance, indicates a “blood stasis” pattern. Both formulas are used for an “interior” pattern; however, we found no item with *p* < 0.05 that indicated an “interior” pattern.

Based on this differentiation, we have developed a predictive model, our final model, which fitted the data well. The final model quantified the tacit knowledge of Kampo specialists in selecting an appropriate Kampo formula for dysmenorrhea. During model selection, a subjective symptom—heat intolerance—and an objective finding—BMI—were eliminated from the final model, whereas all three abdominal findings were included in the final model. These results suggest that abdominal findings are important for specialists in selecting a Kampo treatment from among these two candidate formulas.

The selection of the appropriate formula is important in clinical situations. Each formula has specific characteristics and has been studied based on clinical experience. For example, tokishakuyakusan has been studied for its effect on infertility in rats and mice [23–26]. Keishibukuryogan has been studied for its effect on uterine myoma, not only in rats and mice, but also in humans [27–29].

Furthermore, the efficacy of each of these formulas is different from that of their individual crude constituents; thus, the combination of components is important [30]. For instance, tokishakuyakusan consists of six crude components: Japanese Angelica root, peony root, hoelen, Atractylodes rhizome, Alisma rhizome, and Cnidium rhizome. In contrast, keishibukuryogan consists of five crude components: cinnamon bark, peony root, hoelen, peach kernel, and moutan bark. Peony root is one of the crude drugs that tokishakuyakusan and keishibukuryogan have in common. A decoction of peony root has been used to treat many painful or inflammatory conditions, such as cholangitis, bronchiolitis, rhinorrhea, and muscle cramps. It has been reported to have an anticontraction effect, by suppressing the increase of intracellular calcium ion concentration, and anti-inflammatory effects, by inhibiting the production of prostaglandin E2, leukotriene B4, and nitric oxide [31]. However, some studies found that the isolated crude drug did not act as an anticontraction agent on uterine smooth muscle [32, 33].

The present study has some limitations. Our study involved many Kampo specialists, who may vary in their definitions of each finding. Such variations should be standardized with the advent of modern devices that can objectively examine a patient's tongue [34], abdominal wall [35], or pulse [36]. These objective findings will be incorporated into our model in the future to improve data reliability.

Second, clinical efficacy was not considered as part of this model development. More than 80% of our patients improved to at least some degree after Kampo treatment (data not shown), but retrospective validation of efficacy using medical charts was difficult and incomplete. Whether any formula is truly appropriate should be defined only by its carefully assessed efficacy. Moreover, we considered only the two representative Kampo formulas and did not consider other minor formulas. Although we performed a small external validation or our model, we excluded 41.2% of patients who were treated with minor formulas. If we apply our model in a clinical situation, approximately 40% of patients, who were treated with minor formulas, would have been prescribed either of the two major formulas. In the future, the effectiveness and safety of this model in a clinical situation should be evaluated using a prospective study design.

## 5. Conclusions

We compared the subjective symptoms and objective findings between patients who were prescribed either of the two major Kampo formulas used to treat dysmenorrhea (tokishakuyakusan and keishibukuryogan) and used this to develop a model that could predict the selection of either of these formulas for a patient by Kampo specialists. The effectiveness and safety of this model should be validated in prospective trials.

## Supplementary Material

Appendix Table in Comparison of all items about subjective symptoms and objective findings between the TSS and KBG groups.

## Figures and Tables

**Table 1:** Frequently used Kampo formulas in 222 patients with dysmenorrhea.

Formulas	Number
Keishibukuryogan	73
Tokishakuyakusan	67
Kamishoyosan	20
Anchusan	19
Goreisan	18
Saikokeishikankyoto	13
Tokakujokito	10
Yokukansan	8
Saikokaryukotsuboreito	8
Tokikenchuto	7
Jumihaidokuto	6
Shosaikoto	6
Tokishigyakukagoshuyushokyoto	6
Bukuryoingohangekobokuto	6
Byakkokaninjinto	6
Daisaikoto	5
Hochuekkito	5
Shakuyakukanzoto	5
Hangekobokuto	4
Rikkunshito	4
Others (45 kinds of formulas)	60

Total	356

Five patients from the keishibukuryogan group and 7 from the tokishakuyakusan group were excluded from the comparison and model-development analysis (see Figure 1). A total of 127 patients were prescribed 2 or more formulas, and 356 formulas were prescribed in total.

**Table 2:** Baseline characteristics of the patients included in the study.

	Comparison and model- development set May 2008 to March 2013	External validation set April 2013 to December 2015	*p* value
Number of patients	128	40	N/A
Age at consultation			
Mean ± SD	32.8 ± 8.3	35.0 ± 7.0	0.108^*∗*^
Median	33	37	0.129^†^
Range	12–50	22–47	
N/A	0 (0.0)	0 (0.0)	
Age at menarche, years			
Mean ± SD	12.3 ± 1.5	12.7 ± 2.1	0.245^*∗*^
Median	12	12	0.355^†^
Range	9–17	10–21	
N/A	2 (1.6)	1 (2.5)	
Menstrual cycle, days			
Mean ± SD	29.1 ± 4.8	28.2 ± 2.6	0.162^*∗*^
Median	28	28	0.076^†^
Range	16–60	24–40	
N/A	22 (17.2)	9 (22.5)	
Bleeding period, days			
Mean ± SD	5.9 ± 1.6	5.6 ± 1.5	0.366^*∗*^
Median	6	5	0.196^†^
Range	3–14	3–10	
N/A	13 (10.2)	3 (7.5)	
BMI, kg/m^2^			
Mean ± SD	20.8 ± 3.1	20.8 ± 2.8	0.905^*∗*^
Median	20.4	20.6	0.572^†^
Range	15.9–39.8	16.2–31.6	
<18.5 (slim)	27 (21.1)	5 (12.5)	0.259^‡^
≥25 (obese)	11 (8.6)	2 (5.0)	0.735^‡^
N/A	3 (2.3)	0 (0.0)	
Use of OCs			
No	117 (91.4)	31 (77.5)	0.025^‡^
Yes	11 (8.6)	9 (22.5)	
Delivery			
No	116 (90.6)	35 (87.5)	0.556^‡^
Yes	12 (9.4)	5 (12.5)	
Abortion			
No	114 (89.1)	35 (87.5)	0.778^‡^
Yes	14 (10.9)	5 (12.5)	
Diagnosed organic disease			
No	105 (82.0)	25 (62.5)	0.016^‡^
Endometriosis	14 (10.9)	11 (27.5)	0.019^‡^
Adenomyosis	10 (7.8)	10 (25.0)	0.009^‡^
Infertility (primary and secondary)			
No	123 (96.1)	45 (87.5)	0.146^‡^
Yes	5 (3.9)	5 (12.5)	

N/A, not available; BMI, body mass index; OCs, oral contraceptives.

Findings are expressed as mean ± SD, median, range, or number with percentage in parentheses.

*p* values were calculated using ^*∗*^ *t*-test, ^†^Wilcoxon's rank sum test, and ^‡^Fisher's exact test.

**Table 3:** Baseline characteristics of the patients included in the comparison and model-development analysis.

	The comparison and model development set		*p* value	Other formulas
	TSS group	KBG group		
Number of patients	60	68	N/A	86
Age at consultation				
Mean ± SD	33.3 ± 7.9	32.5 ± 8.7	0.595^*∗*^	33.0 ± 8.1
Median	33	33	0.742^†^	33
Range	17–50	12–50		13–49
N/A	0 (0.0)	0 (0.0)		0 (0.0)
Age at menarche, years				
Mean ± SD	12.2 ± 1.5	12.3 ± 1.6	0.753^*∗*^	12.7 ± 1.6
Median	12	12	0.932^†^	12
Range	9–16	9–17		9–17
N/A	0 (0.0)	2 (2.9)		2 (2.3)
Menstrual cycle, days				
Mean ± SD	29.0 ± 5.7	29.3 ± 3.6	0.772^*∗*^	28.9 ± 4.5
Median	28	28	0.771^†^	28
Range	16–60	25–45		17–60
N/A	6 (10.0)	16 (23.5)		10 (11.6)
Bleeding period, days				
Mean ± SD	5.8 ± 1.3	5.9 ± 1.9	0.841^*∗*^	5.5 ± 1.6
Median	6	6	0.747^†^	5
Range	3–9	3–14		3–14
N/A	4 (6.7)	9 (13.2)		7 (8.1)
BMI, kg/m^2^				
Mean ± SD	19.8 ± 2.3	21.6 ± 3.5	0.001^*∗*^	20.6 ± 2.9
Median	19.4	21.0	0.000^†^	20.0
Range	15.9–26.7	17.0–39.8		15.6–30.1
<18.5 (slim)	21 (35.0)	6 (8.8)	0.000^‡^	26 (30.2)
≥25 (obese)	2 (3.3)	9 (13.2)	0.060^‡^	7 (8.1)
N/A	2 (3.3)	1 (1.5)		4 (4.7)
Use of OCs				
No	54 (90.0)	63 (92.6)	0.754^‡^	77 (89.5)
Yes	6 (10.0)	5 (7.4)		9 (10.5)
Delivery				
No	54 (90.0)	62 (91.2)	1^‡^	75 (87.2)
Yes	6 (10.0)	6 (8.8)		11 (12.8)
Abortion				
No	53 (88.3)	61 (89.7)	1^‡^	74 (86.0)
Yes	7 (11.7)	7 (10.3)		12 (14.0)
Diagnosed organic disease				
No	52 (86.7)	53 (77.9)	0.251^‡^	81 (94.2)
Endometriosis	6 (10.0)	8 (11.8)	0.785^‡^	4 (4.7)
Adenomyosis	2 (3.3)	8 (11.8)	0.103^‡^	2 (2.3)
Infertility (primary and secondary)				
No	59 (98.3)	64 (94.1)	0.370^‡^	81 (94.2)
Yes	1 (1.7)	4 (5.9)		5 (5.8)

TSS, tokishakuyakusan; KBG, keishibukuryogan; N/A, not available; BMI, body mass index; OCs, oral contraceptives.

Findings are expressed as mean ± SD, median, range, or number with percentage in parentheses.

*p* values were calculated using ^*∗*^ *t*-test, ^†^Wilcoxon's rank sum test, and ^‡^Fisher's exact test.

**Table 4:** Comparison of subjective symptoms and objective findings between the TSS and KBG groups.

	The comparison and model development set		*p* value
	TSS group (*n* = 60)	KBG group (*n* = 68)	
Subjective symptoms			
Tendency to sweat			
No	52 (86.7)	40 (58.8)	0.001
Yes	8 (13.3)	28 (41.2)	
Heat intolerance			
No	54 (90.0)	50 (73.5)	0.023
Yes	6 (10.0)	18 (26.5)	
Leg numbness			
No	59 (98.3)	59 (86.8)	0.019
Yes	1 (1.7)	9 (13.2)	
Cold sensation in lower back			
No	57 (95.0)	56 (82.4)	0.030
Yes	3 (5.0)	12 (17.6)	
Lightheadedness			
No	30 (50.0)	46 (67.6)	0.049
Yes	30 (50.0)	22 (32.4)	
Objective findings			
Weak abdomen			
No	28 (46.7)	60 (88.2)	0.000
Yes	32 (53.3)	8 (11.8)	
Strong abdomen			
No	58 (96.7)	50 (73.5)	0.003
Yes	2 (3.3)	18 (26.5)	
Paraumbilical tenderness and resistance			
No	31 (51.7)	10 (14.7)	0.000
Yes	29 (48.3)	58 (85.3)	

TSS, tokishakuyakusan; KBG, keishibukuryogan.

Only factors with *p* value < 0.05 were included. Findings are expressed as number with percentage in parentheses. *p* values were calculated using Fisher's exact test.

**Table 5:** Effects of potential predictor variables and predictor variables in the final model.

	Univariate		Multivariate (final model)		
	Estimates	OR (95% CI)	Estimates	OR (95% CI)	*p* value
(Intercept)			−1.356	0.258 (0.075, 0.752)	0.019
Subjective symptoms					
Tendency to sweat	1.515	4.550 (1.945, 11.684)	0.930	2.533 (0.844, 8.210)	0.106
Heat intolerance	1.176	3.240 (1.248, 9.531)			
Leg numbness	2.197	9.000 (1.617, 168.588)	2.448	11.561 (1.844, 228.900)	0.029
Cold sensation in lower back	1.404	4.071 (1.216, 18.571)	1.559	4.752 (0.870, 35.614)	0.095
Lightheadedness	−0.738	0.478 (0.231, 0.974)	−1.019	0.361 (0.128, 0.971)	0.047
Objective findings					
Slim (BMI < 18.5)	−1.716	0.180 (0.061, 0.460)			
Weak abdomen	−2.148	0.117 (0.045, 0.274)	−1.498	0.224 (0.068, 0.666)	0.009
Strong abdomen	2.346	10.440 (2.827, 67.728)	2.077	7.984 (1.732, 60.429)	0.017
Paraumbilical tenderness and resistance	1.825	6.200 (2.753, 14.968)	2.183	8.870 (2.921, 31.644)	0.000

OR, odds ratio; CI, confidence interval; BMI, body mass index.

**Table 6:** Internal and external validation of the final model.

	Accuracy (%)
Internal validation	
Internal estimate	81.2
Cross-validation estimate	80.5
External validation	85.0
