# Supplementary material for: The Difference between the Two Representative Kampo Formulas for Treating Dysmenorrhea: An Observational Study
Source: Evid Based Complement Alternat Med. 2016 Feb 24;2016:3159617. doi: 10.1155/2016/3159617 (PMC4783569; doi:10.1155/2016/3159617)
Supplement: Supplementary file 1 — Appendix Table in Comparison of all items about subjective symptoms and objective findings between the TSS and KBG groups. [file 3159617.f1.docx]

**Appendix** **table.** Comparison of subjective symptoms and objective findings between the TSS and KBG groups

|  | The comparison and Model development set | |  |
| --- | --- | --- | --- |
|  | TSS group  (n = 60) | KBG group  (n = 68) | p-value |
| ***Subjective symptoms*** |  |  |  |
| Appetite |  |  |  |
| Appetite loss |  |  |  |
| No | 55 (91.7) | 64 (94.1) | 0.733 |
| Yes | 5 (8.3) | 4 (5.9) |  |
| Good appetite |  |  |  |
| No | 54 (90.0) | 61 (89.7) | 1 |
| Yes | 6 (10.0) | 7 (10.3) |  |
| Speed of the meal |  |  |  |
| Slow speed of the meal |  |  |  |
| No | 52 (86.7) | 59 (86.8) | 1 |
| Yes | 8 (13.3) | 9 (13.2) |  |
| Fast speed of the meal |  |  |  |
| No | 48 (80.0) | 51 (75.0) | 0.533 |
| Yes | 12 (20.0) | 17 (25.0) |  |
| Difficulty falling asleep |  |  |  |
| No | 43 (71.7) | 47 (69.1) | 0.847 |
| Yes | 17 (28.3) | 21 (30.9) |  |
| Arousal during sleep |  |  |  |
| No | 46 (76.7) | 44 (64.7) | 0.176 |
| Yes | 14 (23.3) | 24 (35.3) |  |
| Early-morning awakening |  |  |  |
| No | 46 (76.7) | 56 (82.4) | 0.511 |
| Yes | 14 (23.3) | 12 (17.6) |  |
| I dream frequently |  |  |  |
| No | 36 (60.0) | 42 (61.8) | 0.858 |
| Yes | 24 (40.0) | 26 (38.2) |  |
| Single dose of urine |  |  |  |
| Single dose of urine large |  |  |  |
| No | 59 (98.3) | 65 (95.6) | 0.622 |
| Yes | 1 (1.7) | 3 (4.4) |  |
| Single dose of urine low |  |  |  |
| No | 55 (91.7) | 65 (95.6) | 0.473 |
| Yes | 5 (8.3) | 3 (4.4) |  |
| Difficulty urinating |  |  |  |
| No | 59 (98.3) | 66 (97.1) | 1 |
| Yes | 1 (1.7) | 2 (2.9) |  |
| Urination pain |  |  |  |
| No | 59 (98.3) | 65 (95.6) | 0.622 |
| Yes | 1 (1.7) | 3 (4.4) |  |
| Urine leakage |  |  |  |
| No | 60 (100) | 67 (98.5) | 1 |
| Yes | 0 (0.0) | 1 (1.5) |  |
| Enuresis |  |  |  |
| No | 60 (100) | 68 (100) | 1 |
| Yes | 0 (0.0) | 0 (0.0) |  |
| Hard stool |  |  |  |
| No | 51 (85.0) | 59 (86.8) | 0.804 |
| Yes | 9 (15.0) | 9 (13.2) |  |
| Small and round stool |  |  |  |
| No | 53 (88.3) | 59 (86.8) | 1 |
| Yes | 7 (11.7) | 9 (13.2) |  |
| Soft stool |  |  |  |
| No | 43 (71.7) | 50 (73.5) | 0.845 |
| Yes | 17 (28.3) | 18 (26.5) |  |
| Diarrhea |  |  |  |
| No | 47 (78.3) | 56 (82.4) | 0.657 |
| Yes | 13 (21.7) | 12 (17.6) |  |
| Hard to stool |  |  |  |
| No | 56 (93.3) | 59 (86.8) | 0.254 |
| Yes | 4 (6.7) | 9 (13.2) |  |
| Hemorrhoid |  |  |  |
| No | 56 (93.3) | 59 (86.8) | 0.254 |
| Yes | 4 (6.7) | 9 (13.2) |  |
| Anal prolapse |  |  |  |
| No | 60 (100) | 66 (97.1) | 0.498 |
| Yes | 0 (0.0) | 2 (2.9) |  |
| Bloody stool |  |  |  |
| No | 59 (98.3) | 64 (94.1) | 0.370 |
| Yes | 1 (1.7) | 4 (5.9) |  |
| Taking laxatives |  |  |  |
| No | 55 (91.7) | 66 (97.1) | 0.251 |
| Yes | 5 (8.3) | 2 (2.9) |  |
| Depressed mood |  |  |  |
| No | 33 (55.0) | 34 (50.0) | 0.599 |
| Yes | 27 (45.0) | 34 (50.0) |  |
| Forgetfulness |  |  |  |
| No | 48 (80.0) | 53 (77.9) | 0.831 |
| Yes | 12 (20.0) | 15 (22.1) |  |
| Irritated |  |  |  |
| No | 31 (51.7) | 38 (55.9) | 0.723 |
| Yes | 29 (48.3) | 30 (44.1) |  |
| Dry skin |  |  |  |
| No | 34 (56.7) | 37 (54.4) | 0.859 |
| Yes | 26 (43.3) | 31 (45.6) |  |
| Itchy skin |  |  |  |
| No | 33 (55.0) | 46 (67.6) | 0.150 |
| Yes | 27 (45.0) | 22 (32.4) |  |
| Acne |  |  |  |
| No | 42 (70.0) | 42 (61.8) | 0.356 |
| Yes | 18 (30.0) | 26 (38.2) |  |
| Blot |  |  |  |
| No | 51 (85.0) | 50 (73.5) | 0.132 |
| Yes | 9 (15.0) | 18 (26.5) |  |
| Urticaria |  |  |  |
| No | 47 (78.3) | 52 (76.5) | 0.835 |
| Yes | 13 (21.7) | 16 (23.5) |  |
| Wart |  |  |  |
| No | 57 (95.0) | 67 (98.5) | 0.340 |
| Yes | 3 (5.0) | 1 (1.5) |  |
| Athlete's foot |  |  |  |
| No | 60 (100) | 65 (95.6) | 0.247 |
| Yes | 0 (0.0) | 3 (4.4) |  |
| Brittle nails |  |  |  |
| No | 44 (73.3) | 55 (80.9) | 0.398 |
| Yes | 16 (26.7) | 13 (19.1) |  |
| Get tired easily |  |  |  |
| No | 13 (21.7) | 14 (20.6) | 1 |
| Yes | 47 (78.3) | 54 (79.4) |  |
| **Quickness to sweat** |  |  |  |
| No | 52 (86.7) | 40 (58.8) | **0.001** |
| Yes | 8 (13.3) | 28 (41.2) |  |
| Night sweats |  |  |  |
| No | 56 (93.3) | 59 (86.8) | 0.254 |
| Yes | 4 (6.7) | 9 (13.2) |  |
| Hot flush |  |  |  |
| No | 49 (81.7) | 58 (85.3) | 0.637 |
| Yes | 11 (18.3) | 10 (14.7) |  |
| **Heat intolerance** |  |  |  |
| No | 54 (90.0) | 50 (73.5) | **0.023** |
| Yes | 6 (10.0) | 18 (26.5) |  |
| Cold intolerance |  |  |  |
| No | 30 (50.0) | 32 (47.1) | 0.859 |
| Yes | 30 (50.0) | 36 (52.9) |  |
| Attenuation of sexual desire |  |  |  |
| No | 57 (95.0) | 63 (92.6) | 0.722 |
| Yes | 3 (5.0) | 5 (7.4) |  |
| Impotence |  |  |  |
| No | 60 (100) | 68 (100) | 1 |
| Yes | 0 (0.0) | 0 (0.0) |  |
| Neck stiffness |  |  |  |
| No | 16 (26.7) | 14 (20.6) | 0.531 |
| Yes | 44 (73.3) | 54 (79.4) |  |
| Shoulder stiffness |  |  |  |
| No | 11 (18.3) | 10 (14.7) | 0.637 |
| Yes | 49 (81.7) | 58 (85.3) |  |
| Back stiffness |  |  |  |
| No | 42 (70.0) | 37 (54.4) | 0.101 |
| Yes | 18 (30.0) | 31 (45.6) |  |
| Lower back stiffness |  |  |  |
| No | 38 (63.3) | 36 (52.9) | 0.283 |
| Yes | 22 (36.7) | 32 (47.1) |  |
| Facial pain |  |  |  |
| No | 59 (98.3) | 67 (98.5) | 1 |
| Yes | 1 (1.7) | 1 (1.5) |  |
| Hand pain |  |  |  |
| No | 56 (93.3) | 61 (89.7) | 0.540 |
| Yes | 4 (6.7) | 7 (10.3) |  |
| Foot pain |  |  |  |
| No | 53 (88.3) | 54 (79.4) | 0.233 |
| Yes | 7 (11.7) | 14 (20.6) |  |
| Shoulder pain |  |  |  |
| No | 53 (88.3) | 55 (80.9) | 0.331 |
| Yes | 7 (11.7) | 13 (19.1) |  |
| Back pain |  |  |  |
| No | 56 (93.3) | 61 (89.7) | 0.540 |
| Yes | 4 (6.7) | 7 (10.3) |  |
| Hip pain |  |  |  |
| No | 46 (76.7) | 48 (70.6) | 0.548 |
| Yes | 14 (23.3) | 20 (29.4) |  |
| Knee pain |  |  |  |
| No | 54 (90.0) | 60 (88.2) | 0.785 |
| Yes | 6 (10.0) | 8 (11.8) |  |
| Numbness face |  |  |  |
| No | 60 (100) | 66 (97.1) | 0.498 |
| Yes | 0 (0.0) | 2 (2.9) |  |
| Numbness hands |  |  |  |
| No | 57 (95.0) | 60 (88.2) | 0.216 |
| Yes | 3 (5.0) | 8 (11.8) |  |
| **Numbness legs** |  |  |  |
| No | 59 (98.3) | 59 (86.8) | **0.019** |
| Yes | 1 (1.7) | 9 (13.2) |  |
| Numbness back |  |  |  |
| No | 60 (100) | 66 (97.1) | 0.498 |
| Yes | 0 (0.0) | 2 (2.9) |  |
| Trembling face |  |  |  |
| No | 60 (100) | 67 (98.5) | 1 |
| Yes | 0 (0.0) | 1 (1.5) |  |
| Trembling hands |  |  |  |
| No | 57 (95.0) | 60 (88.2) | 0.216 |
| Yes | 3 (5.0) | 8 (11.8) |  |
| Trembling legs |  |  |  |
| No | 60 (100) | 68 (100) | 1 |
| Yes | 0 (0.0) | 0 (0.0) |  |
| Cold sensation in general |  |  |  |
| No | 44 (73.3) | 51 (75.0) | 0.842 |
| Yes | 16 (26.7) | 17 (25.0) |  |
| Cold sensation in hands |  |  |  |
| No | 43 (71.7) | 45 (66.2) | 0.569 |
| Yes | 17 (28.3) | 23 (33.8) |  |
| Cold sensation in legs |  |  |  |
| No | 30 (50.0) | 28 (41.2) | 0.375 |
| Yes | 30 (50.0) | 40 (58.8) |  |
| **Cold sensation in lower back** |  |  |  |
| No | 57 (95.0) | 56 (82.4) | **0.030** |
| Yes | 3 (5.0) | 12 (17.6) |  |
| Heat face |  |  |  |
| No | 49 (81.7) | 46 (67.6) | 0.105 |
| Yes | 11 (18.3) | 22 (32.4) |  |
| Heat hands |  |  |  |
| No | 59 (98.3) | 66 (97.1) | 1 |
| Yes | 1 (1.7) | 2 (2.9) |  |
| Heat legs |  |  |  |
| No | 60 (100) | 67 (98.5) | 1 |
| Yes | 0 (0.0) | 1 (1.5) |  |
| Edema face |  |  |  |
| No | 43 (71.7) | 50 (73.5) | 0.845 |
| Yes | 17 (28.3) | 18 (26.5) |  |
| Edema hands |  |  |  |
| No | 50 (83.3) | 58 (85.3) | 0.811 |
| Yes | 10 (16.7) | 10 (14.7) |  |
| Edema legs |  |  |  |
| No | 34 (56.7) | 30 (44.1) | 0.215 |
| Yes | 26 (43.3) | 38 (55.9) |  |
| Headache |  |  |  |
| No | 24 (40.0) | 29 (42.6) | 0.858 |
| Yes | 36 (60.0) | 39 (57.4) |  |
| Sluggishness |  |  |  |
| No | 49 (81.7) | 49 (72.1) | 0.217 |
| Yes | 11 (18.3) | 19 (27.9) |  |
| Vertigo |  |  |  |
| No | 37 (61.7) | 46 (67.6) | 0.578 |
| Yes | 23 (38.3) | 22 (32.4) |  |
| **Lightheadedness** |  |  |  |
| No | 30 (50.0) | 46 (67.6) | **0.049** |
| Yes | 30 (50.0) | 22 (32.4) |  |
| Dandruff |  |  |  |
| No | 55 (91.7) | 62 (91.2) | 1 |
| Yes | 5 (8.3) | 6 (8.8) |  |
| Hair loss |  |  |  |
| No | 46 (76.7) | 51 (75.0) | 0.840 |
| Yes | 14 (23.3) | 17 (25.0) |  |
| Decreased visual acuity |  |  |  |
| No | 50 (83.3) | 55 (80.9) | 0.819 |
| Yes | 10 (16.7) | 13 (19.1) |  |
| Eyestrain |  |  |  |
| No | 21 (35.0) | 26 (38.2) | 0.718 |
| Yes | 39 (65.0) | 42 (61.8) |  |
| Blurred vision |  |  |  |
| No | 51 (85.0) | 52 (76.5) | 0.268 |
| Yes | 9 (15.0) | 16 (23.5) |  |
| Bleary eyes |  |  |  |
| No | 47 (78.3) | 52 (76.5) | 0.835 |
| Yes | 13 (21.7) | 16 (23.5) |  |
| Dark circles under eyes |  |  |  |
| No | 46 (76.7) | 46 (67.6) | 0.325 |
| Yes | 14 (23.3) | 22 (32.4) |  |
| Sneezing |  |  |  |
| No | 47 (78.3) | 53 (77.9) | 1 |
| Yes | 13 (21.7) | 15 (22.1) |  |
| Nasal discharge |  |  |  |
| White nasal discharge |  |  |  |
| No | 51 (85.0) | 59 (86.8) | 0.804 |
| Yes | 9 (15.0) | 9 (13.2) |  |
| Yellow nasal discharge |  |  |  |
| No | 59 (98.3) | 68 (100) | 0.469 |
| Yes | 1 (1.7) | 0 (0.0) |  |
| Post nasal drip |  |  |  |
| No | 55 (91.7) | 62 (91.2) | 1 |
| Yes | 5 (8.3) | 6 (8.8) |  |
| Stuffy nose |  |  |  |
| No | 46 (76.7) | 54 (79.4) | 0.831 |
| Yes | 14 (23.3) | 14 (20.6) |  |
| Nosebleed |  |  |  |
| No | 56 (93.3) | 66 (97.1) | 0.418 |
| Yes | 4 (6.7) | 2 (2.9) |  |
| Mouth bitter |  |  |  |
| No | 59 (98.3) | 67 (98.5) | 1 |
| Yes | 1 (1.7) | 1 (1.5) |  |
| Saliva comes out |  |  |  |
| No | 60 (100) | 66 (97.1) | 0.498 |
| Yes | 0 (0.0) | 2 (2.9) |  |
| Throat pain |  |  |  |
| No | 52 (86.7) | 58 (85.3) | 1 |
| Yes | 8 (13.3) | 10 (14.7) |  |
| Throat jams |  |  |  |
| No | 53 (88.3) | 61 (89.7) | 1 |
| Yes | 7 (11.7) | 7 (10.3) |  |
| Thirsty |  |  |  |
| No | 48 (80.0) | 52 (76.5) | 0.673 |
| Yes | 12 (20.0) | 16 (23.5) |  |
| Dry mouth |  |  |  |
| No | 49 (81.7) | 57 (83.8) | 0.817 |
| Yes | 11 (18.3) | 11 (16.2) |  |
| Dry lips |  |  |  |
| No | 46 (76.7) | 56 (82.4) | 0.511 |
| Yes | 14 (23.3) | 12 (17.6) |  |
| Take water often |  |  |  |
| No | 37 (61.7) | 51 (75.0) | 0.128 |
| Yes | 23 (38.3) | 17 (25.0) |  |
| Tinnitus |  |  |  |
| No | 49 (81.7) | 49 (72.1) | 0.217 |
| Yes | 11 (18.3) | 19 (27.9) |  |
| Hearing loss |  |  |  |
| No | 58 (96.7) | 63 (92.6) | 0.447 |
| Yes | 2 (3.3) | 5 (7.4) |  |
| Cough |  |  |  |
| No | 58 (96.7) | 65 (95.6) | 1 |
| Yes | 2 (3.3) | 3 (4.4) |  |
| Sputum |  |  |  |
| White sputum |  |  |  |
| No | 57 (95.0) | 65 (95.6) | 1 |
| Yes | 3 (5.0) | 3 (4.4) |  |
| Yellow sputum |  |  |  |
| No | 58 (96.7) | 65 (95.6) | 1 |
| Yes | 2 (3.3) | 3 (4.4) |  |
| Asthma |  |  |  |
| No | 57 (95.0) | 63 (92.6) | 0.722 |
| Yes | 3 (5.0) | 5 (7.4) |  |
| Shortness of breath |  |  |  |
| No | 55 (91.7) | 61 (89.7) | 0.769 |
| Yes | 5 (8.3) | 7 (10.3) |  |
| Palpitation |  |  |  |
| No | 51 (85.0) | 55 (80.9) | 0.641 |
| Yes | 9 (15.0) | 13 (19.1) |  |
| Chest pain |  |  |  |
| No | 53 (88.3) | 64 (94.1) | 0.346 |
| Yes | 7 (11.7) | 4 (5.9) |  |
| Burp |  |  |  |
| No | 55 (91.7) | 62 (91.2) | 1 |
| Yes | 5 (8.3) | 6 (8.8) |  |
| Heartburn |  |  |  |
| No | 52 (86.7) | 59 (86.8) | 1 |
| Yes | 8 (13.3) | 9 (13.2) |  |
| Epigastric jamming discomfort |  |  |  |
| No | 57 (95.0) | 63 (92.6) | 0.722 |
| Yes | 3 (5.0) | 5 (7.4) |  |
| Nausea |  |  |  |
| No | 54 (90.0) | 57 (83.8) | 0.435 |
| Yes | 6 (10.0) | 11 (16.2) |  |
| Vomiting |  |  |  |
| No | 56 (93.3) | 67 (98.5) | 0.185 |
| Yes | 4 (6.7) | 1 (1.5) |  |
| Motion sickness |  |  |  |
| No | 44 (73.3) | 52 (76.5) | 0.689 |
| Yes | 16 (26.7) | 16 (23.5) |  |
| Stomach fullness |  |  |  |
| No | 44 (73.3) | 48 (70.6) | 0.844 |
| Yes | 16 (26.7) | 20 (29.4) |  |
| Stomach rumbling |  |  |  |
| No | 49 (81.7) | 55 (80.9) | 1 |
| Yes | 11 (18.3) | 13 (19.1) |  |
| Flatulence |  |  |  |
| No | 42 (70.0) | 47 (69.1) | 1 |
| Yes | 18 (30.0) | 21 (30.9) |  |
| Sleepy after eating |  |  |  |
| No | 41 (68.3) | 42 (61.8) | 0.464 |
| Yes | 19 (31.7) | 26 (38.2) |  |
| Abdominal pain fasting |  |  |  |
| No | 56 (93.3) | 65 (95.6) | 0.705 |
| Yes | 4 (6.7) | 3 (4.4) |  |
| Abdominal pain after eating |  |  |  |
| No | 54 (90.0) | 62 (91.2) | 1 |
| Yes | 6 (10.0) | 6 (8.8) |  |
| Abdominal pain at upper |  |  |  |
| No | 59 (98.3) | 66 (97.1) | 1 |
| Yes | 1 (1.7) | 2 (2.9) |  |
| Abdominal pain at lower |  |  |  |
| No | 50 (83.3) | 56 (82.4) | 1 |
| Yes | 10 (16.7) | 12 (17.6) |  |
| Hand stiffness |  |  |  |
| No | 59 (98.3) | 62 (91.2) | 0.120 |
| Yes | 1 (1.7) | 6 (8.8) |  |
| Lower extremities weakness |  |  |  |
| No | 58 (96.7) | 66 (97.1) | 1 |
| Yes | 2 (3.3) | 2 (2.9) |  |
| Legs fluctuate |  |  |  |
| No | 52 (86.7) | 63 (92.6) | 0.380 |
| Yes | 8 (13.3) | 5 (7.4) |  |
| Leg spasms |  |  |  |
| No | 49 (81.7) | 50 (73.5) | 0.298 |
| Yes | 11 (18.3) | 18 (26.5) |  |
| Frost bite |  |  |  |
| No | 55 (91.7) | 67 (98.5) | 0.098 |
| Yes | 5 (8.3) | 1 (1.5) |  |
| Menstrual flow |  |  |  |
| Heavy menstrual flow |  |  |  |
| No | 41 (68.3) | 47 (69.1) | 1 |
| Yes | 19 (31.7) | 21 (30.9) |  |
| Less menstrual flow |  |  |  |
| No | 55 (91.7) | 60 (88.2) | 0.571 |
| Yes | 5 (8.3) | 8 (11.8) |  |
| Menstruation textile |  |  |  |
| No | 27 (45.0) | 31 (45.6) | 1 |
| Yes | 33 (55.0) | 37 (54.4) |  |
| Menstrual pain |  |  |  |
| No | 4 (6.7) | 7 (10.3) | 0.540 |
| Yes | 56 (93.3) | 61 (89.7) |  |
| Irregular menstruation |  |  |  |
| No | 39 (65.0) | 38 (55.9) | 0.366 |
| Yes | 21 (35.0) | 30 (44.1) |  |
| Delivery |  |  |  |
| No | 54 (90.0) | 61 (89.7) | 1 |
| Yes | 6 (10.0) | 7 (10.3) |  |
| Spontaneous abortion |  |  |  |
| No | 56 (93.3) | 66 (97.1) | 0.418 |
| Yes | 4 (6.7) | 2 (2.9) |  |
| Induced abortion |  |  |  |
| No | 57 (95.0) | 62 (91.2) | 0.500 |
| Yes | 3 (5.0) | 6 (8.8) |  |
| Pregnancy toxemia |  |  |  |
| No | 60 (100) | 68 (100) | 1 |
| Yes | 0 (0.0) | 0 (0.0) |  |
| Metrorrhagia (Abnormal vaginal bleeding) |  |  |  |
| No | 55 (91.7) | 59 (86.8) | 0.411 |
| Yes | 5 (8.3) | 9 (13.2) |  |
|  |  |  |  |
| ***Objective findings*** |  |  |  |
| **Weak abdomen** |  |  |  |
| No | 28 (46.7) | 60 (88.2) | **0.000** |
| Yes | 32 (53.3) | 8 (11.8) |  |
| **Strong abdomen** |  |  |  |
| No | 58 (96.7) | 50 (73.5) | **0.003** |
| Yes | 2 (3.3) | 18 (26.5) |  |
| Abdominal distension |  |  |  |
| No | 60 (100) | 64 (94.1) | 0.122 |
| Yes | 0 (0.0) | 4 (5.9) |  |
| Splashing sound in the epigastric region |  |  |  |
| No | 56 (93.3) | 67 (98.5) | 0.185 |
| Yes | 4 (6.7) | 1 (1.5) |  |
| Epigastric discomfort |  |  |  |
| No | 51 (85.0) | 53 (77.9) | 0.368 |
| Yes | 9 (15.0) | 15 (22.1) |  |
| Hypochondrial resistance and discomfort |  |  |  |
| No | 31 (51.7) | 32 (47.1) | 0.723 |
| Yes | 29 (48.3) | 36 (52.9) |  |
| Palpable abdominal aortic pulsation |  |  |  |
| No | 34 (56.7) | 49 (72.1) | 0.095 |
| Yes | 26 (43.3) | 19 (27.9) |  |
| Rectus muscle tension |  |  |  |
| No | 53 (88.3) | 62 (91.2) | 0.771 |
| Yes | 7 (11.7) | 6 (8.8) |  |
| Weakness of lower abdomen |  |  |  |
| No | 52 (86.7) | 61 (89.7) | 0.784 |
| Yes | 8 (13.3) | 7 (10.3) |  |
| **Para-umbilical tenderness and resistance** |  |  |  |
| No | 31 (51.7) | 10 (14.7) | **0.000** |
| Yes | 29 (48.3) | 58 (85.3) |  |
| Teeth marks on the edges of the patient's tongue |  |  |  |
| No | 13 (21.7) | 25 (36.8) | 0.0521 |
| Yes | 47 (78.3) | 40 (58.8) |  |
| NA | 0 (0.0) | 3 (4.4) |  |
| Dilatation of the sublingual veins |  |  |  |
| No | 30 (50.0) | 30 (44.1) | 0.722 |
| Yes | 30 (50.0) | 35 (51.5) |  |
| NA | 0 (0.0) | 3 (4.4) |  |

Abbreviations: TSS, tokishakuyakusan; KBG, keishibukuryogan.

Findings are expressed as number with percentage in parentheses. p-values were calculated using Fisher’s exact test.
